# Recombinant human thrombopoietin improves hematopoietic stem cell differentiation and T-cell immune homeostasis in patients with severe aplastic anemia by upregulating c-MPL

**DOI:** 10.3389/fphar.2025.1542837

**Published:** 2025-08-25

**Authors:** Ling Deng, Chenchen Liu, Yiyu Guo, Chunyan Liu, Rong Fu

**Affiliations:** ^1^ Department of Hematology, Tianjin Medical University General Hospital, Tianjin, China; ^2^ Tianjin Key Laboratory of Bone Marrow Failure and Malignant Hemopoietic Clone Control, Tianjin, China; ^3^ Tianjin Institute of Hematology, Tianjin, China; ^4^ Department of Hematology, Tianjin Medical University General Hospital Airport Hospital, Tianjin, China

**Keywords:** severe aplastic anemia, rhTPO, c-MPL, T-cell immune homeostasis, hematopoietic stem cells

## Abstract

**Background:**

Recombinant human thrombopoietin (rhTPO) regulates platelet production by promoting megakaryocyte proliferation and has shown promising therapeutic effects in hematopoietic recovery for severe aplastic anemia (SAA). However, its potential impact on immune cells remains unclear.

**Methods:**

This study included 23 patients with SAA, who were divided into two groups based on whether they received rhTPO. Flow cytometry was used to assess the proportions of peripheral immune cells and hematopoietic stem cells (HSCs), as well as their c-MPL expression. Further validation was performed by *in vitro* culture experiments and SAA mice.

**Results:**

The rhTPO group exhibited an upward trend in platelet counts (PLT), as well as a higher proportion of peripheral CD4^+^ T cells and an increased CD4^+^/CD8^+^ T cell ratio. The expression of the receptor of rhTPO, c-MPL, was significantly increased on CD4^+^ T cells and regulatory T cells (Tregs). More important is we found c-MPL expression on bone marrow CD34^+^ cells was unregulated in the rhTPO group. *In vitro* stimulation of bone marrow mononuclear cells from patients with SAA using rhTPO elevated the proportion of Tregs and the CD4^+^/CD8^+^ T cell ratio. Furthermore, CsA combined with rhTPO treatment in SAA mice significantly restored the proportion of peripheral Tregs.

**Conclusion:**

rhTPO can induce the upregulation of c-MPL expression on HSCs, CD4^+^ T cells, and Tregs in patients with SAA. It accelerates platelet production and regulates the proliferation of CD4^+^ T cells and Tregs, thereby promoting immune homeostasis restoration in SAA.

## Introduction

Severe aplastic anemia (SAA) is an immune-mediated bone marrow failure disorder in which abnormally activated CD8^+^ T cells destroy hematopoietic stem cells (HSCs). Patients with SAA exhibit severe immune dysregulation, with inverted CD4^+^/CD8^+^ T cell ratio, significantly elevated CD8^+^ T cells, and increased secretion of cytokines such as IFN-γ and TNF-α. For transfusion-dependent non-severe aplastic anemia (TD-NSAA) patients and young SAA/very severe aplastic anemia (VSAA) patients without HLA-matched donors, immunosuppressive therapy (IST), consisting of antithymocyte globulin (ATG)/antilymphocyte globulin (ALG) combined with cyclosporine A (CsA), remains the standard first-line treatment (Patel et al., 2022; Peffault De et al., 2022). The primary role of IST is to suppress T cells, reduce the secretion of negative hematopoietic regulatory factors, and decrease bone marrow hematopoietic suppression and destruction, thereby facilitating hematopoietic recovery (Liu and Shao, 2018). Since not all patients receiving IST achieve full hematopoietic recovery, combined hematopoietic support therapy is crucial before the full effects of immunosuppression are realized.

Recent studies have shown that combining IST with androgens, granulocyte colony-stimulating factor (G-CSF), erythropoietin (EPO), or IL-11 can reduce early mortality, infection risk, and bone marrow suppression in patients with SAA, offering a treatment option for those with impaired organ function (Shao et al., 1998; Tichelli et al., 2011). Platelet recovery and maintenance remain major challenges in the treatment of SAA, as platelet counts (PLT) typically take longer to recover and are the first to decline sharply during disease relapse. Recombinant human thrombopoietin (rhTPO) achieves good clinical efficacy in the treatment of SAA (Wang et al., 2015; Zhang et al., 2020; Yang et al., 2024). Thrombopoietin (TPO) binding to its receptor, c-MPL, activates downstream signaling pathways, stimulates the differentiation of bone marrow HSCs into megakaryocytes, promotes megakaryocyte proliferation, and increases the platelet production (Guo et al., 2018). Treatment of SAA with rhTPO or in combination with other hematopoietic factors markedly increases the megakaryocytes, promotes bone marrow recovery, shortens the duration of transfusion dependency, and improves the hematologic response while preventing increases in the incidences of clonal evolution and myelofibrosis (Wang et al., 2015), which suggests a potential therapeutic role of rhTPO as an adjuvant therapy in the treatment of SAA.

Patients with SAA have extremely low residual hematopoiesis in the bone marrow, a significantly reduced trilineage hematopoietic cell counts in the peripheral blood, and a slow clearance of TPO, resulting in a marked elevation of endogenous TPO levels (Zhao et al., 2018). But why the administration of supraphysiological doses of rhTPO, in addition to already significantly increased endogenous TPO levels, still improves hematopoiesis in patients with SAA needs to be further investigated. In this study, we found that rhTPO not only stimulated the recovery of bone marrow HSCs but also affected the immunological status in patients with SAA.

## Materials and methods

### Patients

This study included 23 patients with SAA who were diagnosed according to the standard criteria (Killick et al., 2016) in our hospital between June 2021 and December 2022. The inclusion criteria were as follows: all patients receiving IST had no HLA-matched donor or were not suitable candidates for hematopoietic stem cell transplantation; alanine transaminase (ALT) < 69 U/L, aspartate transaminase (AST) < 46 U/L, total bilirubin <33.0 μmol/L, and serum creatinine <133 μmol/L prior to treatment with rhTPO; there was no history of other severe autoimmune diseases, malignancies, anemia-related heart disease, or thrombotic/embolic events. This study was approved by the Ethics Committee of Tianjin Medical University General Hospital, and all participants provided written informed consent.

All patients were received with standard IST: ALG (Genzyme Polyclonals S.A.S., France; 5 mg/kg/d for 5 consecutive days, intravenous) and CsA (plasma concentration at 200–400 ng/mL, for at least 1 year), or only CsA treatment. Among them, 14 patients were treated with the subcutaneous injection of rhTPO (15,000  U, 3 times a week, 3SBIO, Shenyang, China), until PLT returned to the normal range (100–300 × 10^9^/L). The remaining 9 patients who did not receive rhTPO served as the control group. Platelet transfusions were administered when PLT <20 × 10^9^/L, red blood cell transfusions were given when hemoglobin (Hb) < 60 g/L, and subcutaneous G-CSF was administered when the absolute neutrophil count (ANC) ≤ 0.5 × 10^9^/L. Clinical efficacy was evaluated at 3 or 6 months based on peripheral blood counts (Camitta, 2000). Baseline clinical characteristics of all patients are in Table 1.

**TABLE 1:** Baseline characteristics.

Characteristics	All (n = 23)	rhTPO group (n = 14)	Control group (n = 9)	*p*
Age (years)	50 (18–72)	59 (18–72)	49 (21–70)	0.403
Gender (male/female)	11/12	8/6	3/6	0.491
Severity				0.572
SAA	15	8	7	
VSAA	8	6	2	
Immunosuppressor				0.205
IST	10	6	4	
CsA	13	8	5	
At diagnosis^a^				
Hb (g/L)		69.5 (58.75, 83)	65 (40, 85)	0.892
PLT (×10^9^/L)		14.5 (8.75, 19.75)	37 (12.5, 47.5)	0.120
ANC (×10^9^/L)		0.625 (0.14, 3.11)	0.7 (0.25, 2.35)	0.862
Ret (×10^9^/L)		21.95 (9.25, 63.4)	24.85 (13.65, 65)	0.640

^a^ Peripheral blood cell counts at diagnosis are presented as median (range). Ret, absolute reticulocyte count.

### SAA mice

SAA mice were established as previously described (Ding et al., 2020; Chen et al., 2007). 8-Week-old CB6F1 mice were used as recipients and subjected to 4 Gy total body irradiation (TBI). 3 × 10^6^ lymph nodes cells of 8-week-old C57BL/6 mice (B6, H2^b/b^) and intravenously injected into the recipients via the retro-orbital sinus. The untreated CB6F1 mice were served as the normal control group (NC), while the TBI group received irradiation only. All mice were purchased from Shanghai Southern Model Biotechnology Co., Ltd. The animal experimental protocol was approved by the Animal Welfare and Ethics Committee of Tianjin Medical University General Hospital.

SAA mice were divided into four treatment groups: CsA, rhTPO, CsA combined with rhTPO, and saline control group. CsA was diluted in IMDM medium and administered via intraperitoneal injection at a dose of 50 mg/kg/day for 10 days. rhTPO was administered subcutaneously at 25 μg/kg/day for 10 days. On day 7, peripheral blood cell counts were measured by blood collection via the retro-orbital sinus. All mice were sacrificed on day 14 by cervical dislocation, and knee joints were collected and fixed in 4% paraformaldehyde, followed by decalcification. Tissues were embedded in paraffin, sectioned, and stained with hematoxylin and eosin (H&E) for bone marrow hematopoiesis observation under a microscope. At least 4-5 mice were included in each group, and the experiment was repeated at least three times.

### Cell culture

Bone marrow mononuclear cells (BMNCs) were isolated from bone marrow aspirates of 15 patients with SAA at diagnosed. After separation by density gradient centrifugation, the cells were equally divided into two groups and cultured in DMEM complete medium containing 10% fetal bovine serum. The intervention group was treated with rhTPO (50 ng/mL) and incubated at 37°C and 5% CO_2_ for 48 h. The control group was treated with an equal volume of phosphate buffer solution (PBS).

### Flow cytometry

Fresh peripheral blood from patients with SAA was anticoagulated with EDTA, and bone marrow aspirates were anticoagulated with heparin sodium. Lymphocyte subsets were detected using the BD Multitest IMK Kit (BD Biosciences, San Jose, CA, United States) according to the manufacturer’s instructions. Antibodies used to assess bone marrow immune cells and c-MPL expression included: FITC-CD34, FITC-CD3, PE-CD4, FITC-CD8, FITC-CD25, PE-CD127, PerCP-CD110 (c-MPL), and the corresponding isotype control antibodies for each monoclonal antibody. For the analysis of c-MPL, blood samples were consistently run at a medium flow rate. For the analysis of peripheral blood lymphocytes in SAA mice, the following antibodies were used: PE-Cy7-CD3, APC-CD4, PerCP-CD8, FITC-CD25, PE-CD127 and the corresponding isotype control antibodies. Flow cytometry was performed using the FACSCanto II flow cytometer and analyzed by FlowJo 10.0. All reagents and instruments were purchased from BD Biosciences (San Jose, CA, United States). The plasma cytokine levels (IL-2, IL-4, IL-6, IL-10, IFN-γ, TNF-α and IL-17) were measured using an NMPA-approved kit (Human Th1/Th2 Subset Detection Kit, Hangzhou CellGer Biotechnology Co., Ltd.) via the multiplex bead-based flow fluorescence assay (FACSCanto II) according to the manufacturer’s instructions.

### Statistics

Continuous variables were expressed as median (range) and analyzed using the Wilcoxon signed-rank test. Normally distributed data were presented as mean ± standard deviation (SD) and analyzed using Student’s t-tests, One-way ANOVA and Two-way ANOVA. Bonferroni correction was applied to *p*-values for multiple hypothesis testing. Categorical variables were presented as n (%) and analyzed using the chi-square test. All statistical analyses were performed using GraphPad Prism 8.0 (GraphPad Software, San Diego, CA, United States). *p* < 0.05 was considered statistically significant.

## Results

### rhTPO may induce upregulation of c-MPL expression on HSCs and CD4^+^ T cells in patients with SAA

**FIGURE 1:**
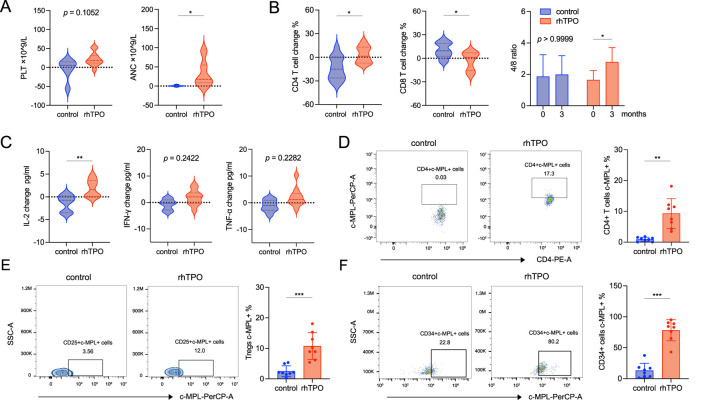
rhTPO may induce upregulation of c-MPL expression on HSCs and CD4^+^ T cells in patients with SAA. **(A)** The differences in changes of PLT (left) and ANC (right) between the control and rhTPO groups from baseline to 3 months of treatment. rhTPO group, n = 14; control group, n = 9. **(B)** Flow cytometry was used to assess the differences between the control and rhTPO groups in the changes of CD4^+^ T (left) and CD8^+^ T cells (median) subset proportions from baseline to 3 months of treatment. The CD4^+^/CD8^+^ T cell ratio (right) from baseline to 3 months of treatment was followed by Two-way ANOVA and Bonferroni correction. mean ± SD, analyzed in duplicates. rhTPO group, n = 14; control group, n = 9. **(C)** Flow cytometry was used to evaluate the differences between the control and rhTPO groups in the changes of plasma cytokines IL-2 (left), IFN-γ (median) and TNF-α (right) from baseline to 3 months of treatment. rhTPO group, n = 14; control group, n = 9. **(D)** The expression of c-MPL on peripheral blood CD4^+^ T cells in control and rhTPO groups after 3 months of treatment, representative FACS plots (left) and summary (right, n = 8, mean ± SD, analyzed in duplicates). **(E)** The expression of c-MPL on peripheral blood Tregs in control and rhTPO groups after 3 months of treatment, representative FACS plots (left) and summary (right, n = 8, mean ± SD, analyzed in duplicates). **(F)** The expression of c-MPL on bone marrow CD34^+^ cells in control and rhTPO groups after 3 months of treatment, representative FACS plots (left) and summary (right, n = 8, mean ± SD, analyzed in duplicates). Bonferroni correction was applied for multiple comparisons where significance is reported as adjusted *p* < 0.05. *, adjusted *p* < 0.05; **, adjusted *p* < 0.01; ***, adjusted *p* < 0.001.

We compared the changes in the peripheral blood cell count, lymphocyte subsets, and cytokine levels from baseline to 3 months between the two patient groups. The PLT levels in the rhTPO group had an upward trend compared with the control group. ANC also showed a moderate increase (adjusted *p* = 0.012), while Hb and Ret remained largely unchanged (Figure 1A; Supplementary Table S1). In lymphocyte subsets, the proportion of CD4^+^ T cells was increased (adjusted *p* = 0.0308) and the proportion of CD8^+^ T cells was decreased (adjusted *p* = 0.0348) in the rhTPO group, resulting in a significant rise in the CD4^+^/CD8^+^ T cell ratio (adjusted *p* = 0.0328). But there is no significance in the proportion of NK cells and B cells (Figure 1B; Supplementary Figure S1A; Supplementary Table S2). In addition, compared with the control group, the rhTPO group showed a slight elevation of IL-2 level in plasma (adjusted *p* = 0.0091), while IFN-γ, TNF-α, IL-4, IL-6, IL-10 and IL-17 levels showed no significant differences (Figure 1C; Supplementary Figure S1B). To further explore the potential mechanisms underlying changes in T cell subset proportions, we assayed the expression of the TPO receptor c-MPL on T cell subsets and HSCs. At 3 months of treatment, the c-MPL expression on CD4^+^ T cells (9.3% ± 4.84% vs. 0.92% ± 0.66%, adjusted *p* = 0.0012) and regulatory T cells (Tregs) (10.77% ± 4.4% vs. 2.516% ± 1.77%, adjusted *p* = 0.0008) in the rhTPO group was significantly higher than that in the control group, but showed no significant change on CD8^+^ T cells (Figures 1D,E; Supplementary Figures S1C, S2, S3A). Interestingly, although the proportion of bone marrow CD34^+^ cells (HSCs) did not differ significantly between the two groups, the c-MPL expression on the bone marrow CD34^+^ cells was significantly increased in the rhTPO group (78.29% ± 17.01% vs. 13.13% ± 11.73%, adjusted *p* < 0.0004) (Figure 1F; Supplementary Figure S1D, S3B).

We evaluated the clinical efficacy. At 3 months, the rate of favorable hematologic response (complete response (CR) + partial response (PR)) showed no significant difference (*p* = 0.742). At 6 months, the rhTPO group demonstrated an increased rate of favorable hematologic response but still without statistically significant (*p* = 0.524) (Supplementary Table S3). We also assessed transfusion independence in both groups. At 1 month, 2 patients in the rhTPO group became independent of platelet and red blood cell transfusions, compared to 1 patient in the control group (*p* = 0.825). By 2 months, 8 patients in the rhTPO group had achieved transfusion independence, compared to only 2 in the control group; however, the difference remained statistically nonsignificant (*p* = 0.099) (Supplementary Table S4). All patients were followed for 12 months. During follow-up, all patients in the rhTPO group were found to be well-tolerated, with no thrombotic events or other significant adverse effects observed. One patient in each group died during months 8–9 of follow-up, both due to severe pulmonary infection secondary to SAA, leading to heart failure and respiratory failure. No patients developed reticulin fibrosis of the bone marrow after 6 months. At 12 months, none of the surviving patients had clonal karyotype or disease progression.

### *In vitro* experiments and SAA mice demonstrate that rhTPO may promote the restoration of CD4^+^ T cell proportions

**FIGURE 2:**
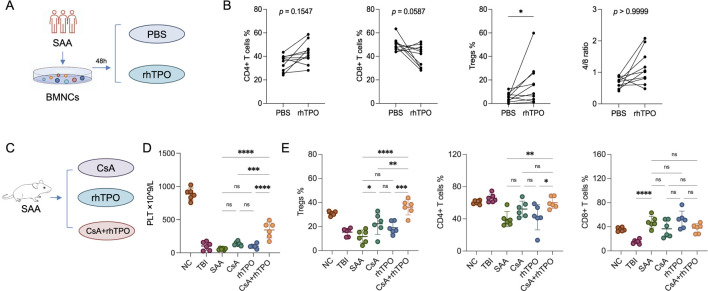
rhTPO may promote the restoration of CD4^+^ T cell proportions. **(A)** Schematic diagram of the *in vitro* experiments. **(B)** Flow cytometry was performed to assay the proportions of T cell subsets (CD4^+^ T cells, CD8^+^ T cells, Tregs, and CD4+/CD8+ T cell ratios) in bone marrow mononuclear cells (BMNCs) from SAA patients after 48 h of *in vitro* stimulation with rhTPO and PBS, respectively. Statistical analyses were performed using two-way ANOVA and followed by Bonferroni correction. n = 10; mean ± SD, analyzed in duplicates. **(C)** Schematic representation of the treatment regimen in SAA mice. **(D)** PLT changes in SAA mice among different treatment groups were analyzed using one-way ANOVA followed by Bonferroni correction. n = 6; mean ± SD, analyzed in duplicates. NC, normal control group; TBI, total body irradiation; SAA, SAA mice treated with saline. **(E)** Flow cytometry was performed to assay the proportions of peripheral T cell subsets (Tregs, CD4^+^T cells and CD8^+^T cells, respectively) in mice subjected to different treatments, which were analyzed using one-way ANOVA followed by Bonferroni correction. n = 6; mean ± SD, analyzed in duplicates. Bonferroni correction was applied for multiple comparisons where significance is reported as adjusted *p* < 0.05. *, adjusted *p* < 0.05; **, adjusted *p* < 0.01; ***, adjusted *p* < 0.001; ****, adjusted *p* < 0.0001; ns, no significance. NC, normal control group; TBI, total body irradiation; SAA, SAA mice treated with saline.

Bone marrow mononuclear cells from patients with SAA were cultured *in vitro* and stimulated with rhTPO for 48 h. There was a significant increase in the proportion of Tregs (Figures 2A,B; Supplementary Table S5). In SAA mice, the group treated with CsA and rhTPO had higher PLT levels and megakaryocyte counts than those treated with CsA or rhTPO alone (Table 2). In addition, the proportion of peripheral Tregs in the CsA + rhTPO group was significantly elevated than that in the CsA group (adjusted *p* = 0.003), but there were no significant differences in the proportion of CD4^+^ and CD8^+^ T cells (Figures 2C,D; Supplementary Tables S6, S7).

**TABLE 2:** SAA mice bone marrow cytology.

No.	Treatment	Megakaryocyte	BM Cellularity (%)	M: E ratio
10	CsA + rhTPO	Normal range	70	Increased
14	CsA + rhTPO	Normal range	70	Normal range
3	CsA	Normal range	50	Normal range
5	CsA	Scarce	70	Slightly increased
40	rhTPO	Scarce	50	Slightly increased
8	rhTPO	Only one	40	Increased
9	SAA	No observed	40	Slightly increased
15	TBI	Normal range	80	Slightly increased
19	NC	Normal range	80	Normal range

BM, bone marrow; M: E ratio, myeloid: erythroid ratio; SAA, SAA mice treated with saline; TBI, total body irradiation; NC, normal control group.

## Discussion

SAA is a bone marrow failure disorder characterized by pancytopenia, commonly caused by hyperactive CD8^+^ T cells targeting HSCs. While IST achieves hematologic responses in 60%–75% of patients, recovery of bone marrow function largely depends on the quantity and functionality of residual HSCs. Our previous studies have shown that combining IST with hematopoietic growth factors significantly improves hematologic response and reduces infection in patients with SAA (Shao et al., 1998). In addition, rhTPO has been shown to enhance hematologic responses and promote bone marrow recovery in patients receiving IST and, in combination with CsA, accelerates platelet recovery (Wang et al., 2015). In this study, we demonstrated that rhTPO combined with CsA significantly increases platelet production and, to some extent, reduces the duration of transfusion dependence.

TPO, primarily produced by the liver, is an endogenous hematopoietic regulator that drives megakaryocyte differentiation and maturation and regulates platelet production. By binding to its receptor c-MPL, TPO activates downstream signaling pathways including JAK/STAT, PI3K/AKT, and RAS/MAPK, stimulating all stages of thrombopoiesis (Guo et al., 2018; Hitchcock et al., 2021). Moreover, the TPO/c-MPL axis plays a critical role in the proliferation and differentiation of HSCs. Mice deficient in TPO or c-MPL exhibit markedly reduced HSCs, which can be reversed by administration of physiological levels of TPO (Zeigler et al., 1994; Wang et al., 2005; Ishikawa et al., 2018). Under normal conditions, c-MPL is highly expressed on hematopoietic stem and progenitor cells, with flow cytometry indicating that approximately 30%–50% of CD34^+^ cells express c-MPL, and even higher expression is observed on the immature CD34^+^CD38^−^ stem cell subset (Ninos et al., 2006). Peripheral B cells typically do not express c-MPL, although a small population of pro-B cells may transiently express it during bone marrow development (Rollinger-Holzinger et al., 1998). Whether resting T cells express c-MPL at the mRNA and protein levels remains poorly studied. Our previous research demonstrated that c-MPL expression was significantly reduced on bone marrow CD34^+^ cells in patients with AA. Autoreactive T cells may suppress c-MPL expression via inflammatory cytokines such as IFN-γ, and the residual CD34^+^ cells show impaired function accompanied by downregulation of c-MPL (Mengying et al., 2023). In this study, we found that CsA combined with rhTPO significantly upregulated c-MPL expression on bone marrow CD34^+^ cells in the patients with SAA, suggesting that rhTPO may not only directly stimulate platelet production but also promote HSCs proliferation and potentially restore their function by inducing c-MPL expression. Although the patients with SAA exhibit high levels of endogenous TPO, the decreased expression of its receptor c-MPL may be responsible for the dysfunction in HSCs proliferation and differentiation.

Previous studies have shown that TPO possesses immunomodulatory effects, such as improving T cell function and inducing the production of related cytokines. TPO has been reported to significantly accelerate thymic T cell reconstitution, particularly CD4^+^ T cells, in mice receiving sublethal irradiation. This was accompanied by improved PLT and faster recovery of erythroid and myeloid lineages (Amado et al., 1998). These findings seem consistent with our results, where CsA combined with rhTPO increased the proportion of peripheral CD4^+^ T cells and Tregs in patients with SAA, along with upregulated c-MPL expression on their surfaces. In contrast, c-MPL expression on CD8^+^ T cells showed no significant change. Tregs regulate the autoimmune microenvironment of SAA by inhibiting overactive T cells (Yan et al., 2015; Kordasti et al., 2016). Therefore, rhTPO may mainly alleviate the immune hyperactivity of SAA by restoring the number of CD4^+^T cells and Tregs. Alvarado et al. demonstrated that an *in vitro* treatment with an increased rhTPO concentration could not abrogate the inhibitory effect of IFN-γ on the self-renewal and proliferation of CD34^+^ cells (Alv et al., 2019). The results of this study showed no significant changes in plasma IFN-γ and TNF-α levels in the rhTPO group, suggesting that rhTPO may have a limited impact on CD8^+^ T cell function.

This study demonstrates that rhTPO not only promotes hematopoiesis but also modulates the immune status in patients with SAA, suggesting that its clinical dosing could be adjusted based on both PLT and immune status. Upon platelet activation, surface of P-selectin is upregulated, promoting binding to leukocytes via P-selectin glycoprotein ligand-1 (PSGL-1) and leading to the formation of leukocyte–platelet aggregates. Conventional flow cytometry has certain limitations in identifying these aggregates, which may result in potential contamination when assessing c-MPL upregulation on lymphocytes. We therefore recommend imaging flow cytometry for a more accurate analysis of rhTPO-induced c-MPL expression on lymphocytes (Hui et al., 2017). And this study has other certain limitations, including a relatively small sample size and a lack of long-term follow-up on immune parameters. Future studies will aim to expand the sample size, extend the follow-up period to assess immune status and disease relapse or progression, and investigate the underlying molecular mechanisms, such as the effects of rhTPO on lymphoid progenitor cell differentiation.

In summary, our results indicated that rhTPO stimulated HSCs recovery and accelerated PLT production by increasing the expression of c-MPL in bone marrow CD34^+^ cells in patients with SAA. In addition, rhTPO was found to exert a major immunomodulatory effect, which promoted the restoration of immune homeostasis in patients with SAA by reversing the imbalance in the CD4^+^/CD8^+^ T-cell ratio, promoting the proliferation of Treg cells, and upregulating c-MPL expression.

## Data Availability

The original contributions presented in the study are included in the article/Supplementary Material, further inquiries can be directed to the corresponding authors.
