# Supplementary material for: Recombinant human thrombopoietin improves hematopoietic stem cell differentiation and T-cell immune homeostasis in patients with severe aplastic anemia by upregulating c-MPL
Source: Front Pharmacol. 2025 Aug 25;16:1542837. doi: 10.3389/fphar.2025.1542837 (PMC12414925; doi:10.3389/fphar.2025.1542837)
Supplement: Supplementary file 1 [file DataSheet1.pdf]

Supplementary Material

Supplementary Table S1. Difference in change in peripheral blood cell count after 3 months of treatment compared with diagnosis [median (range)]

	PLT ($\times 10^9$/L)	Hb (g/L)	ANC ($\times 10^9$/L)	Ret ($\times 10^9$/L)
rhTPO group (n=14)	20 (-7, 54)	10.5 (-31, 80)	3.12 (-0.88, 93.31)	-0.8 (-35.7, 148.8)
Control group (n=9)	2 (-56, 16)	5 (-21, 28)	0.16 (-2.43, 4.10)	28.7 (-8.8, 81.10)
adjusted *p*	0.1052	> 0.9999	0.012	> 0.9999

Bonferroni correction was applied for multiple comparisons, where significance is reported as adjusted $p < 0.05$. PLT, platelet counts; Hb, hemoglobin; ANC, absolute neutrophil count; Ret, absolute reticulocyte count.

Supplementary Table S2. The proportion of peripheral blood lymphocyte subsets after 3 months of treatment compared with diagnosis (mean ± SD)

	rhTPO group (n=14)		Control group (n=9)	
	0 month	3 months	0 month	3 months
$CD4^+$T cells, %	39.45±15.87	41.5±11.12	47.37±13.26	29.10±15.57
$CD8^+$T cells, %	28.49±11.46	25.80±9.89	25.23±6.11	38.57±14.78
B cells, %	9.52±9.79	15.95±9.52	9.78±6.29	5.73±4.20
NK cells, %	19.37±5.49	14.3±9.29	14.18±7.68	23.08±12.59
$CD4^+$/$CD8^+$ T cell ratio	1.86±1.39	1.99±1.20	2.19±1.13	0.96±0.78

Supplementary Table S3. Clinical efficacy [n (%)]

	3 months		6 months	
	CR+PR	NR	CR+PR	NR
rhTPO group (n=14)	1 (7.1)	13 (92.9)	3 (21.4)	11 (78.6)
Control group (n=9)	1 (11.1)	8 (88.9)	1 (11.1)	8 (88.9)
p	0.742		0.524	

CR, complete response; PR, partial response; NR, no response.

Supplementary Table S4. Platelet and red blood cell transfusion independence [n (%)]

	1 month		2 months	
	Independent	Dependent	Independent	Dependent
rhTPO group (n=14)	2 (14.3)	12 (85.7)	8 (57.1)	6 (42.9)
Control group (n=9)	1 (11.1)	8 (88.9)	2 (22.2)	8 (77.8)
p	0.825		0.099	

Supplementary Table S5. The proportion of bone marrow lymphocyte subsets after rhTPO stimulation in vitro (mean ± SD)

	rhTPO group (n=10)	PBS group (n=10)	adjusted *p*
$CD4^+$T cells, %	43.05±9.898	33.62±7.705	0.1547
$CD8^+$T cells, %	40.4±9.168	50.11±5.747	0.0587
Tregs, %	16.65±17.85	5.253±3.684	0.0190
$CD4^+$/$CD8^+$ T cell ratio	1.144±0.5523	0.688±0.1692	> 0.9999

Bonferroni correction was applied for multiple comparisons, where significance is reported as adjusted $p < 0.05$.

Supplementary Table S6. Peripheral blood cell counts in SAA mice on day 7 of treatment (mean ± SD)

group	WBC ($\times 10^9$/L)	RBC ($\times 10^{12}$/L)	Hb (g/L)	PLT ($\times 10^9$/L)
NC	3.858±0.3477	10.12±0.7041	134±2.582	886±90.75
TBI	2.308±1.234	7.718±1.795	111.5±24.19	102.7±62.7
SAA	0.11±0.1245	5.476±0.8369	92.8±18.43	57.5±12.66
CsA	1.342±0.608	8.316±1.618	118.2±25.9	137±29.33
rhTPO	0.3067±0.2688	7.325±1.067	94.17±12.92	99.17±31.21
rhTPO+CsA	2.127±0.6301	9.967±1.214	142.2±7.782	344±119.8

WBC, white blood cell; RBC, red blood cell; Hb, hemoglobin; PLT, platelet; NC, normal control; TBI, total body irradiation; SAA, SAA mice treated with saline.

Supplementary Table S7. Peripheral blood T-cell subsets in SAA mice on day 14 of treatment (mean ± SD)

group	$CD4^+$ T cells %	$CD8^+$ T cells %	Tregs %
NC	60.61±2.363	35.46±3.054	30.98±1.696
TBI	65.64±5.194	15.28±3.521	15.33±3.316
SAA	39.73±9.288	47.66±9.715	11.8±5.388
CsA	52.13±9.817	36.55±14.13	22.34±8.823
rhTPO	42.06±15.72	51.5±14.47	18.94±5.071
rhTPO+CsA	60.04±7.104	37.52±7.818	36.03±6.43

Tregs, regulatory T cells; NC, normal control; TBI, total body irradiation; SAA, SAA mice treated with saline.

Supplementary Figure S1

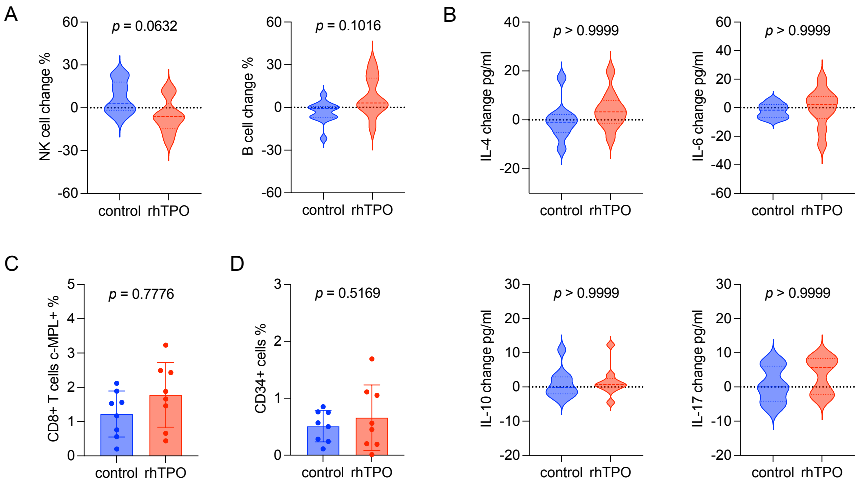

Figure S1. (A) Flow cytometry was used to assess the differences between the control and rhTPO groups in the changes of NK cells (left) and B cells (right) subset proportions from baseline to 3 months of treatment. rhTPO group, n = 14; control group, n = 9. (B) Flow cytometry was used to assess the differences between the control and rhTPO groups in the changes of plasma cytokines IL-4, IL-6 (upper), IL-10 and IL-17 (blow) from baseline to 3 months of treatment. (C) The expression of c-MPL on peripheral blood $CD8^+$ T cells in control and rhTPO groups after 3 months of treatment, representative in summary (n = 8, mean ± SD, analyzed in duplicates). (D) Flow cytometry was used to assess changes in the proportion of bone marrow $CD34^+$ cells in control and rhTPO groups after 3 months of treatment, representative in

summary (n = 8, mean ± SD, analyzed in duplicates). Bonferroni correction was applied for multiple comparisons where significance is reported as adjusted $p < 0.05$.

Supplementary Figure S2

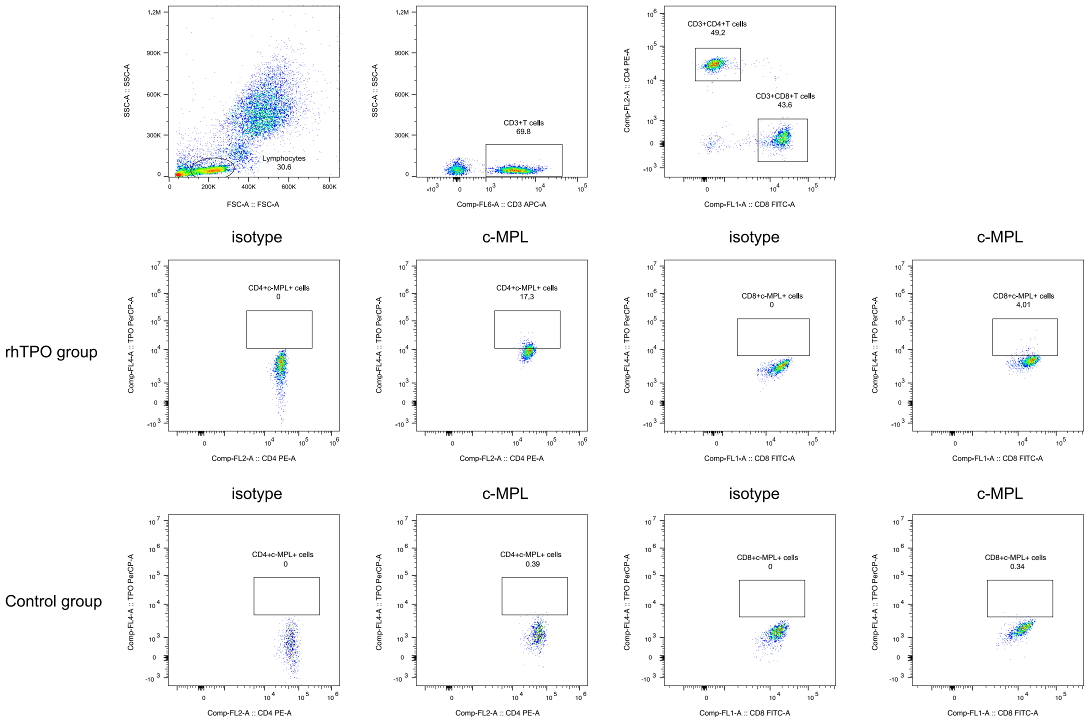

Figure S2. FACS plots illustrating flow cytometry analysis of peripheral blood $CD4^+$ and $CD8^+$ T cells surface c-MPL expression in control and rhTPO groups after 3 months of treatment, including gating strategy and the isotype control.

Supplementary Figure S3

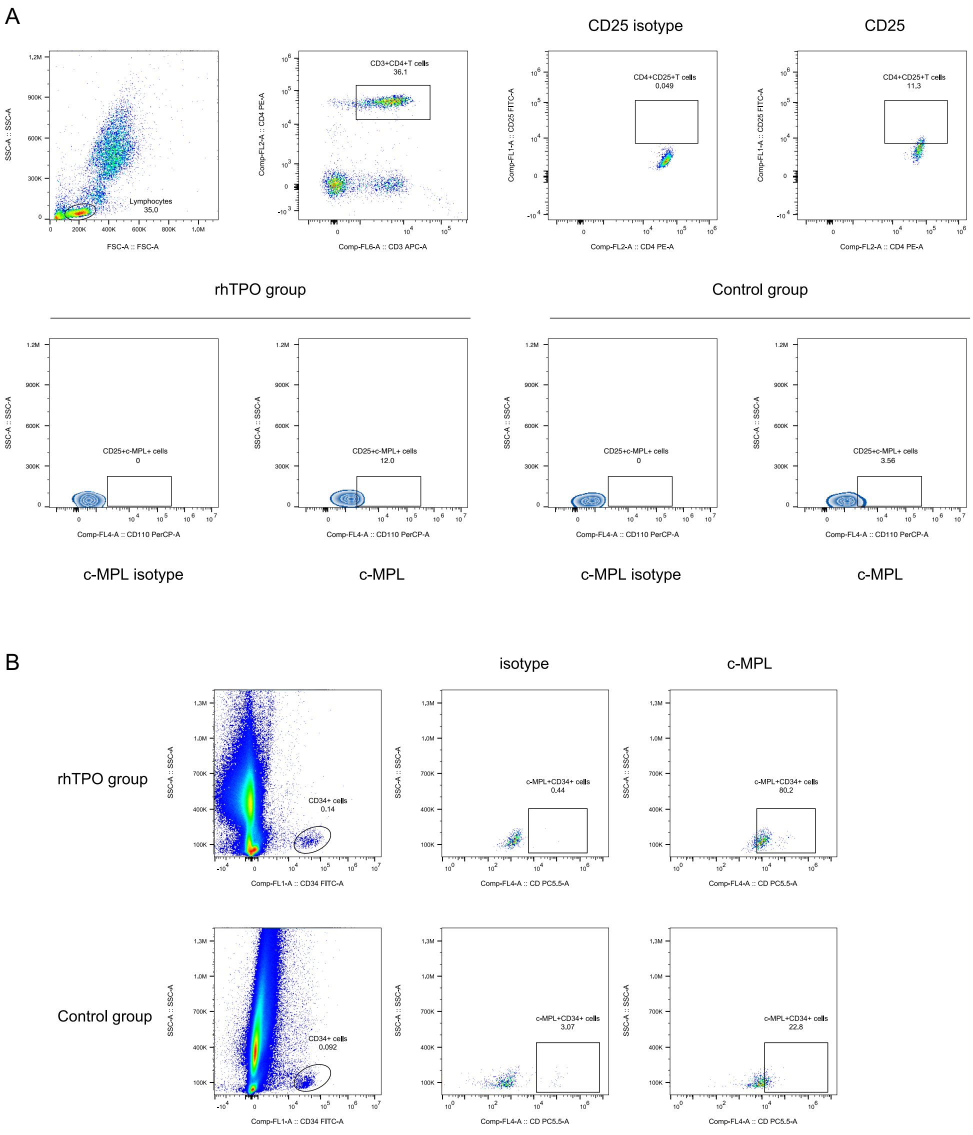

Figure S3. FACS plots illustrating flow cytometry analysis of the c-MPL expression on peripheral blood Tregs (A) and bone marrow CD34$^+$ cells (B) in control and rhTPO groups after 3 months of treatment, including gating strategy and isotype controls.
